# Expression profiling identifies genes involved in emphysema severity

**DOI:** 10.1186/1465-9921-10-81

**Published:** 2009-09-02

**Authors:** Santiyagu M Savarimuthu Francis, Jill E Larsen, Sandra J Pavey, Rayleen V Bowman, Nicholas K Hayward, Kwun M Fong, Ian A Yang

**Affiliations:** 1 Department of Thoracic Medicine, The Prince Charles Hospital, Brisbane, Australia; 2 School of Medicine, The University of Queensland, Brisbane, Australia; 3 Department of human genetics, Oncogenomics Laboratory, Queensland Institute of Medical Research, Brisbane, Australia

## Abstract

Chronic obstructive pulmonary disease (COPD) is a major public health problem. The aim of this study was to identify genes involved in emphysema severity in COPD patients.

Gene expression profiling was performed on total RNA extracted from non-tumor lung tissue from 30 smokers with emphysema. Class comparison analysis based on gas transfer measurement was performed to identify differentially expressed genes. Genes were then selected for technical validation by quantitative reverse transcriptase-PCR (qRT-PCR) if also represented on microarray platforms used in previously published emphysema studies. Genes technically validated advanced to tests of biological replication by qRT-PCR using an independent test set of 62 lung samples.

Class comparison identified 98 differentially expressed genes (*p *< 0.01). Fifty-one of those genes had been previously evaluated in differentiation between normal and severe emphysema lung. qRT-PCR confirmed the direction of change in expression in 29 of the 51 genes and 11 of those validated, remaining significant at *p *< 0.05. Biological replication in an independent cohort confirmed the altered expression of eight genes, with seven genes differentially expressed by greater than 1.3 fold, identifying these as candidate determinants of emphysema severity.

Gene expression profiling of lung from emphysema patients identified seven candidate genes associated with emphysema severity including *COL6A3, SERPINF1*, *ZNHIT6, NEDD4, CDKN2A, NRN1 *and *GSTM3*.

## Introduction

Chronic obstructive pulmonary disease (COPD) is a major health burden worldwide [1]. Smoking is the primary cause of COPD, with up to 50% of smokers developing the disease [2]. It is frequently under-diagnosed and under-treated [3] since its early stages are often asymptomatic. COPD patients are classified into mild, moderate and severe based on the degree of airflow limitation, which is a result of damage in the large airways (bronchitis), small airways (bronchiolitis) and or alveoli (emphysema). Emphysema affects 40% of heavy smokers [4] and causes loss of elastic recoil, leading to abnormal gas exchange and breathlessness. Despite smoking cessation, some individuals continue to deteriorate, developing severe emphysema due to persistent inflammation and continued damage [5]. A recent meta-analysis by Godtfredson *et al *suggests that former smokers with mild to moderate COPD have better morbidity and mortality outcomes [6]. Hence, early identification of susceptible individuals would increase the opportunity for improved intervention, early treatment and prevention of progression. Patho-biological mechanisms in emphysema development include inflammation, protease and antiprotease imbalance and oxidative stress [7], but many pathways, both within and outside of these mechanisms, remain to be explored. In this study we used microarrays to simultaneously study multiple genes with the aim of identifying markers and/or pathways that would enable greater understanding of the biology of emphysema progression in susceptible smokers, and which could have potential as diagnostic tools or therapeutic targets.

High throughput microarray technology has been used to profile gene expression patterns to identify important genes and pathways implicated in chronic lung disease. Susceptibility studies in COPD have used lung tissue and primary cells to profile gene expression. Four of these studies have compared gene expression changes between various Global Initiative for Chronic Obstructive Lung Disease (GOLD) stages (I-IV) [8-11], but only two studies so far have profiled lungs from patients clinically stratified by emphysema (these are discussed in detail below) [12,13].

Spira *et al *[12] performed a case-control study which compared the gene expression profile of 20 smokers with severely emphysematous lungs and 14 smokers with normal or mildly emphysematous lungs [12]. Similarly, Golpon *et al *[13] compared lung expression profiles between controls and patients with either severe emphysema or alpha 1 antitrypsin (α_1_AT) enzyme deficiency [13]. These studies identified differential expression of particular genes as well as a global reduction in gene expression in severe emphysema, compared with normal lung, potentially explained by the relative acellularity of end-stage emphysema. Validation of published expression differences and identification of additional genes responsible for the progression of emphysema would contribute to progress in understanding patho-biology and improving clinical management.

We hypothesised that gene expression profiling would identify differentially expressed genes that are associated with the progression from mild to moderate emphysema. We chose these stages for two main reasons: (i) we considered this phase of progression (from mild to moderate) to be most critical in the development of symptomatic, clinically significant emphysema, as well as more responsive to treatment than end-stage lung disease and (ii) to avoid lack of sensitivity from previously shown global gene downregulation of severe acellular end-stage emphysema. The transcriptome profile in mild and moderately emphysematous lung was therefore compared to identify gene candidates for severity of disease, which were then validated in an independent set of test patients.

## Materials and methods

### Subjects and samples for The Prince Charles Hospital training set

Patients who had undergone curative resection for lung cancer and who agreed to donate resected lung to The Prince Charles Hospital (TPCH) lung tissue bank were selected for this study if they fulfilled the following inclusion criteria: 1) > 20 pack years of self-reported smoking history (where one pack-year was defined as the equivalent of 20 cigarettes per day for one year), 2) ceased smoking > 10 months prior to surgery (to avoid the effects of current smoking on gene expression) and 3) chronic airflow limitation with FEV_1_/VC ratio < 0.70. Exclusion criteria were the following: 1) current use of inhaled or oral steroids (to exclude the effects of steroids on gene expression), 2) pre-operative chest x-ray showing obstructive pneumonitis (to exclude the potential confounding effect of obstructive pneumonitis), 3) α_1_AT deficiency (S or Z alleles) ascertained by genotyping genomic DNA (to exclude the effects of α_1_AT associated emphysema) [14] and 4) other lung pathology causing impaired gas transfer (interstitial lung disease, pulmonary embolism). Thirty cases met criteria for this study. The project was approved by the Human Research Ethics Committees of The University of Queensland and TPCH. All subjects gave written, informed consent prior to the surgery.

All subjects had pre-bronchodilator lung function testing before surgery. Spirometry and gas transfer were performed according to American Thoracic Society standards on the Jaeger Compactlab Transfer and Body Systems (Jaeger, Hoechberg, Germany) and results were compared to predicted values [15,16]. The single breath carbon monoxide diffusing capacity (DLCO) was divided by alveolar volume to estimate carbon monoxide diffusing capacity within the volume of lung accessed by the single breath (KCO). The 30 COPD patients were arbitrarily classed as mild emphysema with KCO ≥ 75% predicted (n = 10) and moderate emphysema with KCO < 75% predicted (n = 20).

### Microarray experiments

Immediately after surgery the non-tumor tissue from the peripheral lung was macroscopically dissected by a pathologist under aseptic conditions, snap-frozen in liquid nitrogen, and stored at -80°C. Total RNA was extracted from these samples using Trizol (Invitrogen Corporation, Carlsbad, CA, USA), DNase treated (Qiagen, Hilden, Germany) and quality checked on an Agilent Bioanalyzer (Agilent Technologies Inc., Santa Clara, CA, USA) as previously published from our laboratory [17]. Lung and universal reference RNA (Stratagene, La Jolla, CA, USA) was reverse transcribed, labeled with Cy5 and Cy3 (Amersham/GE Healthcare, Buckinghamshire, England) respectively and co-hybridized onto a 22K Operon V2.1 Human Genome Oligo Microarray chip http://www.operon.com containing 21,329 70 mer probes representing ~14,200 named transcripts printed by the British Columbia Gene Array Facility http://www.microarray.prostatecentre.com. Study design for microarray experiments conformed to MIAME guidelines http://www.mged.org/Workgroups/MIAME/miame_checklist.html. All data have been deposited in the NCBI Gene Expression Omnibus (GEO) public repository http://www.ncbi.nlm.nih.gov/geo and can be accessed through the accession number GSE17770.

### Microarray data preprocessing

Raw images were imported into Imagene V5.1 (BioDiscovery, Inc., El Segundo, CA, USA) for background correction, filtering of spots with poor morphology, and calculation and extraction of median intensity signals. Avadis V4.3 (Strand Genomics, Bangalore, India), was used to suppress 'bad' spots, which were signals fewer than 20 pixels or greater than 65,000 pixels. Data was centralized across all samples using Lowess normalization, to account for non-linear dye bias. The Cy5/Cy3 ratio was then computed and log transformed to the base two. Genes with log ratio variation of *p *> 0.05 were excluded as their signal ratios displayed no significant variance from the mean signal ratio of the samples.

### Genelist selection and external validation

Class comparison analysis, based on the supervising parameter KCO, was performed in BRB ArrayTools V3.5β1 (developed by Dr Richard Simon and Amy Peng Lam, freely accessible online http://linus.nci.nih.gov/BRB-ArrayTools.html) to identify genes differentially expressed between mild (≥ 75% predicted KCO) and moderate emphysema (<75% predicted KCO) groups categorized by gas transfer.

In order to prioritise significant dysregulated genes for technical validation, we initially selected those represented on the gene expression microarray platforms used in two previously published studies that analyzed emphysematous tissue (Spira *et al *[12] and Golpon *et al *[13]) accessed from Gene Expression Omnibus (GEO) Spira *et al *(GEO series GSE1650) used the Affymetrix HG-U133A gene chip that contained probes for ~22,500 human transcripts and Golpon *et al *(GEO series GSE1122) used the HuGeneFL Affymetrix gene chip that contained probes for ~6,086 transcripts. Chip Comparer http://tenero.duhs.duke.edu/genearray/perl/chip/chipcomparer.pl was used to find genes that were common between the Operon V2.1, Affymetrix HG-U133A and HUGeneFL platforms. We chose to validate by qRT-PCR only those genes represented both in Operon and at least one of the other two platforms. This will facilitate external validation and identification of robust genes involved in the pathogenesis of emphysema.

### Technical validation of mRNA in the training set by quantitative reverse transcriptase PCR (qRT-PCR)

Total RNA prepared for the microarray experiments was reverse transcribed using Superscript III (Invitrogen Technologies, Carlsbad, California) according to the manufacturer's instructions, and 30 ng of cDNA was used for each qRT-PCR reaction. For each candidate gene, forward and reverse primers were designed using Primer Express v1.5 (PerkinElmer, Inc., Wellesley, MA, USA) to a target close to the microarray probe to amplify the same transcripts if applicable. Primer sequences are listed in the additional file (see Additional file 1). SYBR^® ^green chemistry (Applied Biosystems, Foster City, California) [18] was used to measure the mRNA level of the gene of interest on a real time rotary analyzer (Rotor-Gene 6000, Corbett Life Science, NSW, Australia) [19]. Target genes were normalized to the geometric mean of three housekeeping genes - 18S rRNA, alpha actinin 4 (*ACTN4*) and hepatocyte growth factor-regulated tyrosine kinase substrate (*HGS*) [20]. The primer sequences for the housekeepers were 18s fwd: 5'-cggctaccacatccaaggaa-3', rev: 3'-gctggaattaccgcggct-5' ACTN4 fwd: 5'-agcgcaagaccttcacgg-3' rev: 3'-tcatcaatgttctcgatctgtgtg-5' and HGS fwd: 5'-acctgctgaagagacaagtggag-3', rev: 3'-ggtacaggatcttgttacggacgt-5'. The ratio of mean expression in cases with moderate emphysema to the mean expression in cases with mild emphysema was compared between qRT-PCR and microarray signals. Signal ratios of genes demonstrating consistent change in direction of transcript expression in both qRT-PCR and microarray were judged technically validated.

### Biological replication of mRNA in test set

Technically validated candidate genes that were statistically significant (*t*-test, *p *< 0.05) were selected for biological replication on an independent test set of 62 lung samples from the TPCH lung tissue bank. The subjects in the test set included smokers with at least ten pack-years smoking history with mild or moderate emphysema. The test set consisted of 21 patients with mild emphysema (>75% predicted KCO) and 41 patients with moderate emphysema (40-74% predicted KCO). These samples did not overlap with the samples used in the training set. Total RNA was isolated and reverse transcribed to cDNA as described above. Quantitative RT-PCR was performed and the mean expression ratio was calculated. Genes that showed concordant direction of transcript expression in the test and training set were judged biologically validated.

## Results

### Demographics

The demographics of the 30 training set and 62 test set subjects are summarised in Table 1. All subjects in the training set were Caucasian former smokers with >20 pack year smoking history and there were more males than females. The subjects were classified as stage I (mild COPD) (9 subjects, 30%) and stage II (moderate COPD) (21 subjects, 70%) according to GOLD guidelines. For the supervised class comparison, emphysema severity in these COPD patients was classified physiologically by the KCO measurement into mild (n = 10, median 79, range 75-85% predicted) and moderate (n = 20, median 69, range 38-74% predicted) emphysema groups.

**Table 1:** Demographics of TPCH training set (n = 30) and TPCH test set (n = 62)

	TPCH training set		TPCH test set	
	
	Mild emphysema	Moderate emphysema	Mild emphysema	Moderate emphysema
n	10	20	21	41
				
Age (Mean ± SD yrs)	71 ± 4	67 ± 7	63 ± 10	61 ± 10
Range	63-77	53-78	42-78	41-82
Male/Female	8/2	14/6	18/3	23/18
				
FEV_1_%predicted (Mean ± SD)	81 ± 17	68 ± 15	84 ± 11	84 ± 19
Range	52-107	50-97	61-104	45-116
				
FEV1/VC (Mean ± SD) %	60 ± 5	54 ± 14	67 ± 6	65 ± 8
Range	50-70	40-70	55-77	48-80
				
KCO %predicted (Mean ± SD)	79 ± 3	66 ± 9	79 ± 3	62 ± 9
Range	75-85	38-74	75-83	43-74
				
Pack years (Mean ± SD)	62 ± 32	75 ± 49	65 ± 52	48 ± 24
Range	28-135	24-240	26-225	13-108
				
Site of tissue collection*	1-LLL	1-LL	2-LLL	2-LL
	1-LUL	5-LUL	6-LUL	3-LLL
	1-RLL	7-RLL	3-RLL	9-LUL
	1-LL &1-RL	5-RUL	3-RML	1-RL
	5-RUL	2-RML	7-RUL	5-RLL
				4-RML

**GOLD Classification**				

Stage I Mild (≥ 80%)	6	3	13	26
				
Stage II Moderate (≤ 50-80%)	4	17	8	13
				
Stage III Severe (≤ 30-50%)	0	0	0	2

* LL - Left Lung, LLL - Left Lower Lobe, LUL - Left Upper Lobe, RL - Right Lung, RLL - Right Lower Lobe, RUL - Right Upper Lobe, RML - Right Middle Lobe,

All subjects in the test set were Caucasian. Emphysema severity was categorized by KCO as mild emphysema (n = 21, median KCO 79% predicted, range 75-80% predicted) and moderate emphysema (n = 41, median KCO 63% predicted, 43-74% predicted).

### Microarray data analysis

The filtering of poor quality spots and normalisation resulted in a list of 20,274 probes comprising 13,178 known genes. Of these, 6,420 transcripts representing 4,159 known genes varied significantly (*p *< 0.05) from the median expression of all genes, and hence were chosen for gene selection analysis.

### Genelist selection and external validation

**Figure 1:**
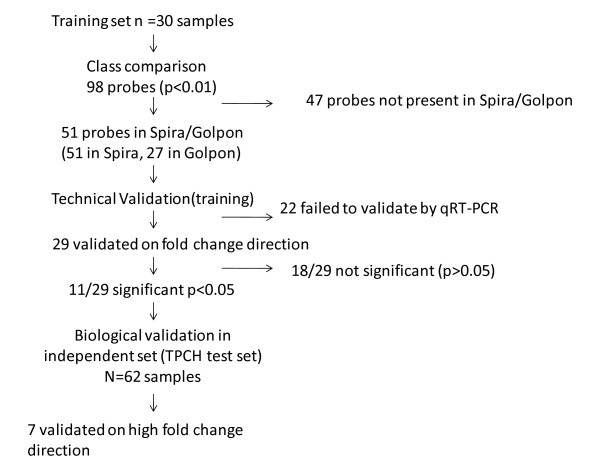
**Flowchart of the study design and outcome**.

Class comparison analysis identified 98 differentially expressed genes (*p *< 0.01) between mild and moderate emphysema (See Additional file 2). Fifty-one of the 98 genes were represented on the arrays (HG-U133A) used in the Spira *et al *study [12] that were used to profile 34 lung tissue samples (20 severe emphysema, 14 mild emphysema/normal lung) and 27 probes were represented in Golpon *et al *(Affymetrix HuGeneFL) study [13] that profiled 10 lung tissue samples with 5 severe emphysema and 5 normal lung. These 27 probes were also represented on the HG-U133A arrays used by Spira *et al*. A flow chart showing prioritisation of genelists and the analysis work flow is included in Figure 1. To test the accuracy of these genes to classify or predict emphysema severity, leave-one out class prediction analysis using the multivariate predictor, Nearest Centroid Correct was used, correcting for random variance, in BRB ArrayTools. The shortlisted 51 genes were 100% accurate (100% sensitivity and 100% specificity) in classifying emphysema severity in the 30 training samples. The classification accuracy of the 51 and 27 probes on the Spira *et al *and Golpon *et al *datasets respectively were 77% (83% sensitivity and 67% specificity) and 80% (80% sensitivity and 80% specificity) in predicting normal and severe emphysema (See Additional file 3). The hierarchical clustering of these 51 genes in TPCH training set is included in as additional File (See Additional file 4).

### Technical validation of mRNA expression using qRT-PCR in the training set

The 51 shortlisted genes progressed to technical validation by qRT-PCR in the training set. For 29 genes the direction of mean expression ratios by qRT-PCR (up- or down-regulation) was concordant with their corresponding microarray expression ratios. Eleven of the 29 genes demonstrated statistically significant differences between mild and moderate emphysema (*t*-test, *p *< 0.05). For information on genes and their *p *values please see Additional file 2.

### Biological replication of mRNA expression in the TPCH test set and *in silico *replication in public test sets

**Figure 2:**
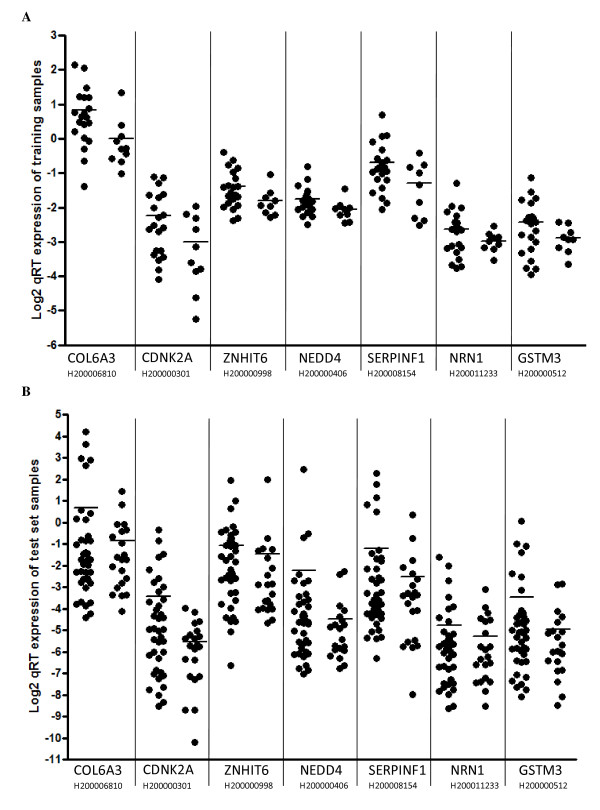
**mRNA expression measured by qRT-PCR of seven candidate genes with greater than 1.3 fold change in TPCH training set (a) (n = 30) and TPCH test set (b) (n = 62)**. The figure shows the average gene expression of the mild (right) and moderate (left) emphysema.

**Figure 3:**
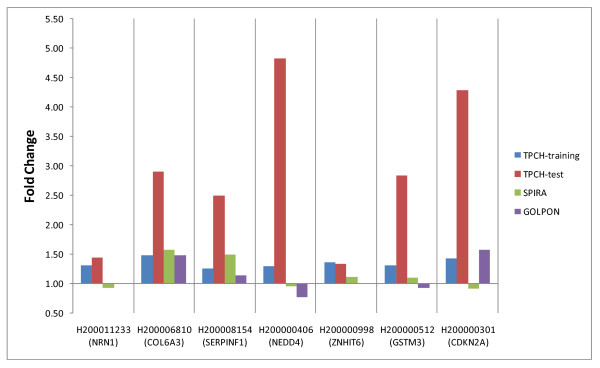
**Comparison of mRNA expression in seven candidate genes between TPCH test (n = 30, microarray) and training set (n = 62 qRT-PCR data), with two public microarray datasets of lung tissue samples (Spira *et al***, [12], **n = 34; and Golpon *et al ***[13], **n = 10)**. Fold change represents mean expression ratio of moderate versus mild emphysema (TPCH training set), severe/mild emphysema versus normal (Spira *et al*), or severe emphysema versus normal samples (Golpon *et al*). The absence of a bar indicates the gene was not represented on the microarray platform.

These 11 genes were submitted to biological replication in a test set of 62 lung samples from the TPCH lung tissue bank. Of the 11 genes selected from microarray analysis and technically validated by qRT-PCR, eight displayed concordant increased or decreased expression. Seven of the genes displayed greater than 1.3 fold changes in expression between moderate versus mild emphysema lung samples in the TPCH test set. These seven candidate emphysema severity genes were 60% (59% sensitive and 62% specific) accurate in classifying mild and moderate emphysema patients in TPCH independent test, 83% (83% sensitive and 83% specific) and 80% (80% sensitive and 80% specific) accurate in classifying normal and severe emphysema patients in Spira and Golpon studies respectively (See Additional file 5). The qRT-PCR expression results of the training and independent test sets are shown in Figure 2a &2b. *In silico *comparison of direction of gene expression between the three studies displayed five of seven genes to be concordant between Spira and TPCH cohort. Three of the five genes common with the HuGeneFL platform were observed to be concordant in direction of expression between the Golpon and TPCH cohorts (Figure 3).

## Discussion

We used gene expression microarrays with subsequent technical, biological and *in silico *validation, to identify genes differentially expressed between mild and moderate emphysema as defined by KCO. We believe that the rigour of this approach minimises the chance of identifying false positive genes and ensures that the most robust candidate genes are selected for functional validation. This study is the first to profile the genes involved in the progression of emphysema by comparing mild and moderate emphysema patients. This stage of disease is more amenable to intervention and therapy, and avoids a low signal to noise issue from the known global gene expression downregulation of severe end stage emphysema.

The 98 genes differentially expressed between mild and moderate emphysema were prioritised for technical validation, initially by choosing 51 genes represented in at least one of two public emphysema microarray platforms (Spira *et al *[12] and Golpon *et al *[13]). Using qRT-PCR, 29 of the 51 genes (56%) passed technical validation in our training set of 30 samples. In contrast to this study, Spira *et al *[12] and Golpon *et al *[13] randomly chose fewer candidate genes to validate by qRT-PCR (a total of ten and three candidate genes, respectively) and they found qRT-PCR expression to correlate strongly with microarray expression. Additionally Spira *et al *reported qRT-PCR expression results on only four samples (two severe and two normal emphysema lungs), whereas we decided to validate on all 30 training samples to avoid selection bias and chance. Nonetheless our lower technical validation rate could also be influenced by differences in platforms (Operon versus Affymetrix), technology (dual versus single channel), oligo printing (spotted versus photolithography) and/or oligo length (70 mer versus 25 mer). Despite these differences, genes with consistent expression differences between mild and moderate emphysema were identified in our study.

To facilitate external validation we used previously published emphysema datasets (Spira *et al *[12] and Golpon *et al *[13]) to verify the expression of our candidate genes. We compared the genes differentially expressed between mild and moderate emphysema at p < 0.01 (n = 98) in our study, with those in Spira *et al *(n = 102) and Golpon *et al *(n = 84) studies. Only two genes, *COL6A3 *and *SERPINF1*, were significantly differentially expressed at p < 0.01 and in the same direction in Spira *et al *and our study. Only one gene, *DOCK2*, was differentially expressed but in different directions in Golpon *et al *and our study. Comparing the Spira *et al *and Golpon *et al *samples, we also identified one gene, *TOMM20*, to be differentially expressed but in different directions. Minimal or no gene overlaps between the three studies is a common observation in array comparisons, and likely to be due to the different populations studied, variation in biology, platforms, bioinformatics, statistical chance and technical differences [17,21]. A recent publication by Zeskind *et al *also emphasizes this issue of low reproducibility of differentially expressed genes between cohorts [22].

**Figure 4:**
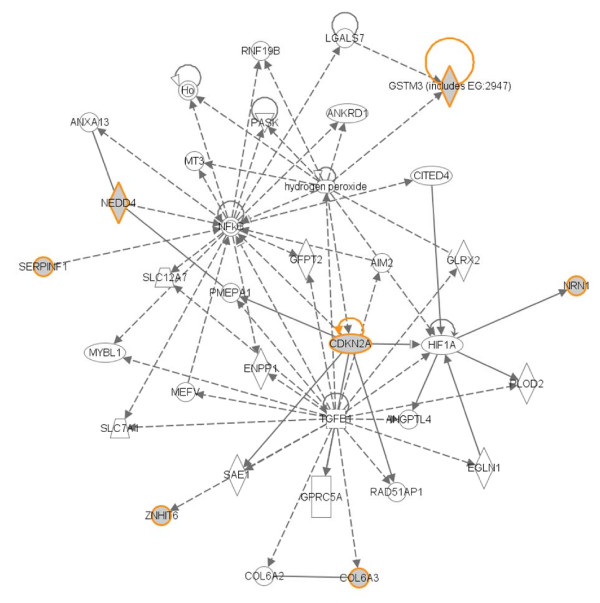
**Ingenuity Pathway Analysis (IPA) on the seven validated candidate genes**. *Bold lines *indicates direct link, *dotted lines *indicate indirect link. Grey nodes indicate input genes into the pathway analysis and the different symbols indicate gene functions. *Horizontal oval = transcription regulator, vertical diamond = enzyme and circle = other*.

To our knowledge, this is the first and only study so far in emphysema to use an independent test cohort to verify the strength of candidate genes. Use of an independent test set for biological validation has been uncommon in previous gene expression profiling studies of emphysema in COPD patients. Eight genes showed concordant change in expression between TPCH training and test sets, and seven of the genes had 1.3 to 4.8 fold change in expression in the moderate emphysema compared with mild emphysema in the TPCH test set, providing increased confidence on the validity of these genes as candidates. The seven genes also showed reasonably high accuracy in classifying normal/mild and moderate/severe emphysema. The candidate genes (*CDKN2A*, *GSTM3*, *COL6A3*, *SERPINF1*, *NRN1*, *NEDD4 *and *ZNHIT6*) had ontologies that were relevant to emphysema progression, including cell cycle regulation (*CDKN2A*) [23], collagen (*COL6A3*) [24], anti-angiogenesis (*SERPINF1*) [25] and oxidative stress (*GSTM3*) [26]. The expressions of all genes were disease associated, except for GSTM3 which was up regulated in the moderate emphysema cases. Few studies have also found an increase in GSTM3 expression in mild/moderate COPD smokers; this strengthens their role as protective intracellular and extracellular lung mediators [27,28]. To evaluate direct and indirect gene networks, we used Ingenuity Pathway Analysis (IPA) (Ingenuity Systems, http://www.ingenuity.com/) to map biological pathways that linked these genes (Figure 4). All eight genes were directly or indirectly linked within one network. For example, *COL6A3 *and the *ZNHIT6 *complex are indirectly regulated by cytokine growth factor, TGFβ1, which is linked directly to the *CDKN2A *complex and indirectly to the NFκB complex. The NFκB complex in turn indirectly regulates the enzymes NEDD4, GSTM3, and SERPINF1. CDKN2A, a cell cycle regulator, has a direct effect on NEDD4 and NRN1 through the PMEPA1 complex and transcriptional regulator HIF1A respectively. Canonical pathway analysis showed other pathways by which these genes could be involved, such as cell cycle checkpoint, p53 signaling, IGF-1 signaling, NRF2 mediated oxidative stress, Wnt/β-Catenin signaling and others (see Additional file 6). The genes were also significantly enriched in ontologies including development, differentiation and enzyme regulation (using DAVID - Database for Annotation, Visualization and Integrated Discovery) (see Additional file 7a &7b) [29]. To clarify the importance of these genes in emphysema progression, further functional characterisation is now required to measure the downstream effects from gene activation or gene inactivation and in *in vitro *or *in vivo *disease models.

A potential limitation of this study is the use of gas transfer measurements (KCO) to classify emphysema severity and lack of histological verification of emphysema severity in the lung samples tested. This was a challenge for this study due to the lack of availability of fresh and formalin fixed paraffin embedded tissue (FFPE) sections from the same site for mRNA analysis and pathological quantification respectively. Despite this, we were able to biologically replicate the expression of candidate genes in an independent set of lung tissues. Also to develop biological markers for disease severity it is important to correlate expression to clinical phenotypes such as KCO and FEV_1_. By correlating gene expression profile with DLCO and FEV_1_, Spira *et al *[12] and Golpon *et al *[13] identified genes significantly associated with emphysema, including oxidative stress, immune, inflammation and extracellular matrix. Despite the TPCH test set being randomly selected, candidate genes still showed similar gene dysregulation to the TPCH training set when stratified by KCO, thus providing reassurance about the robustness of these genes as potential candidates for emphysema severity. Another potential drawback is the prioritisation of our gene list differentiating mild versus moderate emphysema samples using published studies [12,13] that compared normal versus severe emphysema lung samples. Although these were different stages of emphysema, we felt that this was a valid approach to prioritising our gene list for further validation, because we reasoned that involved pathways would be more dysregulated along the continuum of normal, mild, moderate and severe emphysema.

In conclusion, we have used microarray technology to identify seven plausible candidate genes with potential involvement in the progression from mild to moderate emphysema, two of which, *COL6A3 *and *SERPINF1*, are concordantly increased in three different studies. It is highly likely that pathways rather than single genes are involved in progression of emphysema, mandating further investigation of the pathways in which these candidate genes are involved. Future goals include measurement of protein expression and characterization of function by knocking down candidate expression *in vitro *and quantifying cellular endophenotypes relevant to emphysema. These candidates could then be used to develop therapeutic targets against emphysema progression and potential diagnostic biomarkers to identify smokers with mild to moderate emphysema in COPD patients who are most susceptible to disease progression.

## Conclusion

This study reports the identity of seven candidate genes that could be involved in emphysema severity. These genes have been technically and biologically validated in in-house training and independent datasets respectively. In addition, candidate genes also predicted normal and severe emphysema in Spira *et al *and Golpon *et al *datasets with a high accuracy of 83% and 80% respectively. The use of these genes as therapeutic or diagnostic tools warrants further investigation.

## Competing interests

The authors declare that they have no competing interests.

## Authors' contributions

SS: Performed all experiments, data analysis and prepared the manuscript. JL: Optimized the microarray experiments and assisted with microarray data analysis. SP: Provided technical support and assisted in microarray data normalization and analysis. NK: Study design, project plan and data analysis. RB: Study design, project plan and data analysis. KF: Study design, project plan and data analysis. IY: Study design, project plan and data analysis. All authors read and approved the final manuscript.

## Supplementary Material

Additional file 1 **Primer sequences of genes chosen for technical and biological validation**. List of primer sequences used in the validation of microarray probes using qRt-PCR Click here for file

Additional file 2 **Table of 91 genes identified using class comparison analysis**. Genes differentially expressed between mild and moderate emphysema patients. "Y" indicates that the probes have been represented in Affymetrix HG-U133A microarray chip. Click here for file

Additional file 3 **Comparison of class prediction analysis of 51 genes in public datasets**. Class prediction results of 51 genes in TPCH training, Spira and Golpon dataset using Nearest Centroid Correct algorithm. "YES" indicates that the sample has been classified correctly and "NO" indicates that the sample has been classified incorrectly. Click here for file

Additional file 4 **Dendrogram of shortlisted 51 genes**. Supervised two-dimensional hierarchical clustering based on average linkage uncentered correlation of emphysema samples using microarray expression data of the 51 genes represented in Spira and Golpon platforms chosen for qRT-PCR validation on TPCH training set. Each column represents a sample and each row represents a gene. Mild emphysema samples are indicated by the blue bar and moderate emphysema samples are indicated by the orange bar. Heatmap indicates level of gene expression, red, high expression, green, low expression in moderate compared to mild emphysema severity. Click here for file

Additional file 5 **Comparison of class prediction analysis of 7 candidate genes in public datasets**. Class prediction results of 7 genes in TPCH test, Spira and Golpon dataset using Nearest Centroid Correct algorithm. "YES" indicates that the sample has been classified correctly and "NO" indicates that the sample has been classified incorrectly. Click here for file

Additional file 6 **Pathway analysis on candidate genes**. Canonical Pathway analysis in IPA on the seven validated candidate genes. The most significant functional and canonical groups, with *p *< 0.05 are presented. The bars represent p-value in logarithmic scale for each functional or canonical group and genes assigned to each of the functions are listed. Click here for file

Additional file 7 **Over-representation of gene ontologies in candidate genes**. Heatmap (a) and enrichment score (b) of gene ontologies overrepresented in six of the seven candidates. **a) **Represents common gene ontologies enriched in the candidate genes. **b) **Significant clustering (Fisher's Exact, *p *< 0.05) of molecular, biological and cellular functions in the candidate genes. Click here for file

## References

[B1] Pauwels RA Rabe KF Burden and clinical features of chronic obstructive pulmonary disease (COPD) Lancet 2004 364 9434 613 20 10.1016/S0140-6736(04)16855-4 15313363

[B2] Rennard SI Vestbo J COPD: the dangerous underestimate of 15% Lancet 2006 367 9518 1216 9 10.1016/S0140-6736(06)68516-4 16631861

[B3] Takahashi T Ichinose M Inoue H Shirato K Hattori T Takishima T Underdiagnosis and undertreatment of COPD in primary care settings Respirology (Carlton, Vic) 2003 8 4 504 8 10.1046/j.1440-1843.2003.00501.x 14629656

[B4] Hogg JC Wright JL Wiggs BR Coxson HO Opazo Saez A Pare PD Lung structure and function in cigarette smokers Thorax 1994 49 5 473 8 10.1136/thx.49.5.473 8016769 PMC474869

[B5] Willemse BW ten Hacken NH Rutgers B Lesman-Leegte IG Postma DS Timens W Effect of 1-year smoking cessation on airway inflammation in COPD and asymptomatic smokers Eur Respir J 2005 26 5 835 45 10.1183/09031936.05.00108904 16264044

[B6] Godtfredsen NS Lam TH Hansel TT Leon ME Gray N Dresler C COPD-related morbidity and mortality after smoking cessation: status of the evidence Eur Respir J 2008 32 4 844 53 10.1183/09031936.00160007 18827152

[B7] Barnes PJ Shapiro SD Pauwels RA Chronic obstructive pulmonary disease: molecular and cellular mechanisms Eur Respir J 2003 22 4 672 88 10.1183/09031936.03.00040703 14582923

[B8] Bhattacharya S Srisuma S Demeo DL Shapiro SD Bueno R Silverman EK Molecular biomarkers for quantitative and discrete COPD phenotypes American journal of respiratory cell and molecular biology 2009 40 3 359 67 10.1165/rcmb.2008-0114OC 18849563 PMC2645534

[B9] Wang IM Stepaniants S Boie Y Mortimer JR Kennedy B Elliott M Gene expression profiling in patients with chronic obstructive pulmonary disease and lung cancer American journal of respiratory and critical care medicine 2008 177 4 402 11 10.1164/rccm.200703-390OC 17975202

[B10] Ning W Li CJ Kaminski N Feghali-Bostwick CA Alber SM Di YP Comprehensive gene expression profiles reveal pathways related to the pathogenesis of chronic obstructive pulmonary disease PNAS 2004 101 41 14895 900 10.1073/pnas.0401168101 15469929 PMC522001

[B11] Oudijk EJ Nijhuis EH Zwank MD Graaf EA van de Mager HJ Coffer PJ Systemic inflammation in COPD visualised by gene profiling in peripheral blood neutrophils Thorax 2005 60 7 538 44 10.1136/thx.2004.034009 15994259 PMC1747456

[B12] Spira A Beane J Pinto-Plata V Kadar A Liu G Shah V Gene expression profiling of human lung tissue from smokers with severe emphysema AJRCMB 2004 31 6 601 10 15374838 10.1165/rcmb.2004-0273OC

[B13] Golpon HA Coldren CD Zamora MR Cosgrove GP Moore MD Tuder RM Emphysema lung tissue gene expression profiling AJRCMB 2004 31 6 595 600 15284076 10.1165/rcmb.2004-0008OC

[B14] Sandford AJ Weir TD Spinelli JJ Pare PD Z and S mutations of the alpha1-antitrypsin gene and the risk of chronic obstructive pulmonary disease AJRCMB 1999 20 2 287 91 9922220 10.1165/ajrcmb.20.2.3177

[B15] Morris JF Koski A Johnson LC Spirometric standards for healthy nonsmoking adults Am Rev Respir Dis 1971 103 1 57 67 5540840 10.1164/arrd.1971.103.1.57

[B16] Cotes JE Lung Function 1993 5 Blackwell Scientific Publications, London

[B17] Larsen JE Pavey SJ Passmore LH Bowman R Clarke BE Hayward NK Expression profiling defines a recurrence signature in lung squamous cell carcinoma Carcinogenesis 2007 28 3 760 6 10.1093/carcin/bgl207 17082175

[B18] Lentschat A Karahashi H Michelsen KS Thomas LS Zhang W Vogel SN Mastoparan, a G protein agonist peptide, differentially modulates TLR4- and TLR2-mediated signaling in human endothelial cells and murine macrophages J Immunol 2005 174 7 4252 61 15778388 10.4049/jimmunol.174.7.4252

[B19] Pfaffl MW A new mathematical model for relative quantification in real-time RT-PCR Nucleic Acids Res 2001 29 9 e45 10.1093/nar/29.9.e45 11328886 PMC55695

[B20] Vandesompele J De Preter K Pattyn F Poppe B Van Roy N De Paepe A Accurate normalization of real-time quantitative RT-PCR data by geometric averaging of multiple internal control genes Genome Biol 2002 3 7 RESEARCH0034 10.1186/gb-2002-3-7-research0034 12184808 PMC126239

[B21] Verducci JS Melfi VF Lin S Wang Z Roy S Sen CK Microarray analysis of gene expression: considerations in data mining and statistical treatment Physiological genomics 2006 25 3 355 63 10.1152/physiolgenomics.00314.2004 16554544

[B22] Zeskind JE Lenburg ME Spira A Translating the COPD Transcriptome: Insights into Pathogenesis and Tools for Clinical Management Proceedings of the American Thoracic Society 2008 5 8 834 41 10.1513/pats.200807-074TH 19017738 PMC2645236

[B23] Sato M Shames DS Gazdar AF Minna JD A translational view of the molecular pathogenesis of lung cancer J Thorac Oncol 2007 2 4 327 43 17409807 10.1097/01.JTO.0000263718.69320.4c

[B24] Sabatelli P Bonaldo P Lattanzi G Braghetta P Bergamin N Capanni C Collagen VI deficiency affects the organization of fibronectin in the extracellular matrix of cultured fibroblasts Matrix Biol 2001 20 7 475 86 10.1016/S0945-053X(01)00160-3 11691587

[B25] Cosgrove GP Brown KK Schiemann WP Serls AE Parr JE Geraci MW Pigment epithelium-derived factor in idiopathic pulmonary fibrosis: a role in aberrant angiogenesis American journal of respiratory and critical care medicine 2004 170 3 242 51 10.1164/rccm.200308-1151OC 15117744

[B26] Crawford EL Khuder SA Durham SJ Frampton M Utell M Thilly WG Normal bronchial epithelial cell expression of glutathione transferase P1, glutathione transferase M3, and glutathione peroxidase is low in subjects with bronchogenic carcinoma Cancer research 2000 60 6 1609 18 10749130

[B27] Bentley AR Emrani P Cassano PA Genetic variation and gene expression in antioxidant related enzymes and risk of COPD: a systematic review Thorax 2008 63 11 956 61 10.1136/thx.2007.086199 18566111 PMC3032799

[B28] Harju T Mazur W Merikallio H Soini Y Kinnula VL Glutathione-S-transferases in lung and sputum specimens, effects of smoking and COPD severity Respiratory research 2008 9 80 10.1186/1465-9921-9-80 19077292 PMC2654438

[B29] Dennis G Jr Sherman BT Hosack DA Yang J Gao W Lane HC DAVID: Database for Annotation, Visualization, and Integrated Discovery Genome biology 2003 4 5 P3 10.1186/gb-2003-4-5-p3 12734009 PMC193660

