# Supplementary material for: Expression profiling identifies genes involved in emphysema severity
Source: Respir Res. 2009 Sep 2;10(1):81. doi: 10.1186/1465-9921-10-81 (PMC2746189; doi:10.1186/1465-9921-10-81)
Supplement: Additional file 1 — Primer sequences of genes chosen for technical and biological validation. List of primer sequences used in the validation of microarray probes using qRt-PCR [file 1465-9921-10-81-S1.doc]

**Additional file1**

**File Format:** DOC

**Title:** Primer sequences of genes chosen for technical and biological validation.

**Description:**  List of primer sequences used in the validation of microarray probes using qRt-PCR

| Unique ID | Primer sequences | |
| --- | --- | --- |
|  | Forward (5’-3’) | Reverse (5’-3’) |
| H200000301 | ccaacgcaccgaatagttacg | ccatcatcatgacctggatcg |
| H200000406 | ttcatgatatggaatctgtggatagtg | cctgaggtccaattctgttgg |
| H200000512 | aatgccatcttgcgctacatc | agtctcaccacacatgttgtgct |
| H200000945 | cagccagagatcaccagaagc | cgcacgatgttggaatgct |
| H200000998 | ttacatgtggtattgaaaggatcca | tggtagattcactcttcacttggtg |
| H200001000 | taatggaggagctataaattgcacaa | ctaatgatcttgcttcctcttgagtg |
| H200001346 | tctgccacgtaccttgtctctg | ctccgacatgatcctgctgat |
| H200001438 | ggatgagcatggcttacatactaagtac | tgaactcaatctgcatcattcgat |
| H200001736 | gaacaaagtgaattccaaggcag | gcgatccactcggcagtt |
| H200001908 | ggcaagtggaagaagaggagc | cggtaatctttggccgtgac |
| H200002010 | tcttgaagctgccgctg | gcaaggatctgcgactcga |
| H200002632 | ctcagatggtgacaagaagacactc | ggccagcattgtcttgaagaa |
| H200002708 | acggccatacggagatcaga | acgaataactcagtgcaaggca |
| H200002716 | ggacgcaggattggagacc | tgccgagcagtccatacagat |
| H200002856 | ccatacaattggatctcagaccac | gatatcaaccaatcgagtccatctt |
| H200003203 | aggaggagtatcaggcgttcc | ttggtgtcaccaaaggacttgta |
| H200003420 | tcatcaagtggcataacaagctg | ggccacagaatggtcctgaa |
| H200003537 | gggagggaagtctggaagacc | gctgtgtgtctgacggatcttg |
| H200003640 | gaaagagtggattcaactgcacat | tgttgatgatcgagagtcacactg |
| H200004928 | caagttgctaatgctatacaacctgataac | tgggcttcacaagcctgc |
| H200005339 | ggccaaagactacatggagagg | ttcaccaaccaactaggctgc |
| H200005357 | tggttgccaagatatcggatg | aacggagccaaggcctatg |
| H200005448 | atcgtgagaggatggttgcac | agacatctttggactgttcttcacc |
| H200006290 | cacggatcgctgctgtg | tctccagacgtttgtagcaacagt |
| H200006635 | ttgttggccggctacacac | tcccagctccatcacctga |
| H200006810 | aacagatatatgcaagttgccgaa | ccataccagaatcttgcacagc |
| H200007262 | cagaaaaagccaagaatagagatgg | gtatgtcacattgattcacacattcag |
| H200007517 | tcctggaagcatacactacacagatag | ggaggtagagaggcagaaggaga |
| H200007642 | caggaccgacaagtcaagtgc | ggcatccagttaagccagagc |
| H200007867 | atgttagaggcagttcacacaatcc | gatcactgtgaacaatgccatgt |
| H200008070 | tgtaatcacagaacaggtcagagacag | gtacgttccaggattgagtagctg |
| H200008154 | caggagatgaagctgcaatcc | ccacctgagtcagcttgatgg |
| Unique ID | Primer sequences | |
|  | Forward (5’-3’) | Reverse (5’-3’) |
| H200008156 | catatgagtccaacaagcaatgaag | ggttgctgtccgggatttt |
| H200008433 | ccatttccaaggcactggtc | tctattgtggcactgttctcctgt |
| H200008505 | gcaatcgtcatagccagtcgtac | aggcaactgttgactgccttc |
| H200009299 | ccaacggcgatactctctgg | ggttctccttcgataatcttgatctc |
| H200009441 | aacgcagaggatgctcagga | ccacggagtactggtgaaagttc |
| H200010090 | gcactatgtctgcggaagtcc | cttcatttccttctgctcctc |
| H200011233 | gcctggacgacaagacgaac | aaatcctcccagtatgtgcaca |
| H200011369 | atgttcctggcagaccctgtac | caggagcaagtccaagagcg |
| H200011944 | tgaagtaacagacagcatcaactgg | caactacatggtcatcctcaccac |
| H200016317 | ctctggagatctggatgacttgg | gacaacttcagggccgatca |
| H200016590 | gtgactgcataccagaatgataagataac | tcttcggccatttccagtgt |
| H200017197 | agctcatgggaggactagacatg | aagtgttggtgatgtggttgaga |
