# Supplementary material for: Expression profiling identifies genes involved in emphysema severity
Source: Respir Res. 2009 Sep 2;10(1):81. doi: 10.1186/1465-9921-10-81 (PMC2746189; doi:10.1186/1465-9921-10-81)
Supplement: Additional file 2 — Table of 91 genes identified using class comparison analysis. Genes differentially expressed between mild and moderate emphysema patients. "Y" indicates that the probes have been represented in Affymetrix HG-U133A microarray chip. [file 1465-9921-10-81-S2.doc]

**Additional file 2**

**File Format:** DOC

**Title:** Table of 91 genes identified using class comparison analysis.

**Description:** Genes differentially expressed between mild and moderate emphysema patients. “Y” indicates that the probes have been represented in Affymetrix HG-U133A microarray chip.

| **Unique id** | **Spira set** | **Mean array fold change** | **Mean TPCH training fold change** | **Mean TPCH independent set fold change** | **Array Parametric p-value** | **TPCH training p- value** | **TPCH independent set p- value** | **GeneBank accession** | **Gene symbol** |
| --- | --- | --- | --- | --- | --- | --- | --- | --- | --- |
| H200000301 | Y | 1.46 | 1.70 | 4.28 | 0.0022 | 0.0318 | 0.0099 | [NM_000077](http://www.ncbi.nlm.nih.gov/entrez/query.fcgi?db=Nucleotide&term=U26727) | [CDKN2A](http://www.ncbi.nlm.nih.gov/entrez/query.fcgi?cmd=search&db=gene&term=CDKN2A) |
| H200000406 | Y | 1.27 | 1.26 | 4.83 | 0.0071 | 0.0252 | 0.2142 | [D42055](http://www.ncbi.nlm.nih.gov/entrez/query.fcgi?db=Nucleotide&term=AL832063,D42055) | [NEDD4](http://www.ncbi.nlm.nih.gov/entrez/query.fcgi?cmd=search&db=gene&term=NEDD4) |
| H200000512 | Y | 1.29 | 1.41 | 2.84 | 0.0091 | 0.0386 | 0.0646 | [NM_000849](http://www.ncbi.nlm.nih.gov/entrez/query.fcgi?db=Nucleotide&term=CR614504) | [GSTM3](http://www.ncbi.nlm.nih.gov/entrez/query.fcgi?cmd=search&db=gene&term=GSTM3) |
| H200000677 | Y | 0.79 | * |  | 0.0084 |  |  | [NM_000226](http://www.ncbi.nlm.nih.gov/entrez/query.fcgi?db=Nucleotide&term=S69510,Z29074) | [KRT9](http://www.ncbi.nlm.nih.gov/entrez/query.fcgi?cmd=search&db=gene&term=KRT9) |
| H200000945 | Y | 0.76 | 0.95 |  | 0.0025 | 0.7881 |  | [NM_001220](http://www.ncbi.nlm.nih.gov/entrez/query.fcgi?db=Nucleotide&term=U23460) | [CAMK2B](http://www.ncbi.nlm.nih.gov/entrez/query.fcgi?cmd=search&db=gene&term=CAMK2B) |
| H200000998 | Y | 1.39 | 1.37 | 1.33 | 0.0026 | 0.0218 | 0.5769 | [NM_017953](http://www.ncbi.nlm.nih.gov/entrez/query.fcgi?db=Nucleotide&term=AK000767,AL442074) | [ZNHIT6](http://www.ncbi.nlm.nih.gov/entrez/query.fcgi?cmd=search&db=gene&term=NP_060423) |
| H200001000 | Y | 0.63 | 1.19 |  | 0.0023 |  |  | [NM_006903](http://www.ncbi.nlm.nih.gov/entrez/query.fcgi?db=Nucleotide&term=) | [SID6-306](http://www.ncbi.nlm.nih.gov/entrez/query.fcgi?cmd=search&db=gene&term=NP_789843) |
| H200001346 | Y | 0.67 | 1.44 |  | 0.0009 |  |  | [NM_006747](http://www.ncbi.nlm.nih.gov/entrez/query.fcgi?db=Nucleotide&term=) | [SIPA1](http://www.ncbi.nlm.nih.gov/entrez/query.fcgi?cmd=search&db=gene&term=SIPA1) |
| H200001438 | Y | 0.75 | 1.11 |  | 0.0053 |  |  | [AF136407](http://www.ncbi.nlm.nih.gov/entrez/query.fcgi?db=Nucleotide&term=AF136407) | [C6orf5](http://www.ncbi.nlm.nih.gov/entrez/query.fcgi?cmd=search&db=gene&term=C6orf4) |
| H200001736 | Y | 1.36 | 1.14 |  | 0.0038 | 0.4437 |  | [NM_001545](http://www.ncbi.nlm.nih.gov/entrez/query.fcgi?db=Nucleotide&term=X81788) | [ICT1](http://www.ncbi.nlm.nih.gov/entrez/query.fcgi?cmd=search&db=gene&term=ICT1) |
| H200001865 | Y | 0.50 | * |  | 0.0099 |  |  | [AK057328](http://www.ncbi.nlm.nih.gov/entrez/query.fcgi?db=Nucleotide&term=AK057328) |  |
| H200001908 | Y | 0.66 | 1.25 |  | 0.00002 |  |  | [NM_018203](http://www.ncbi.nlm.nih.gov/entrez/query.fcgi?db=Nucleotide&term=AK096590,AK001610) | [FLJ10748](http://www.ncbi.nlm.nih.gov/entrez/query.fcgi?cmd=search&db=gene&term=NP_060673) |
| H200002010 | Y | 1.32 | 1.22 |  | 0.0075 | 0.1160 |  | [U79297](http://www.ncbi.nlm.nih.gov/entrez/query.fcgi?db=Nucleotide&term=U79297,BC035087) |  |
| H200002080 |  | 0.80 |  |  | 0.0063 |  |  | [AF121858](http://www.ncbi.nlm.nih.gov/entrez/query.fcgi?db=Nucleotide&term=AF121858) | [SNX8](http://www.ncbi.nlm.nih.gov/entrez/query.fcgi?cmd=search&db=gene&term=SNX8) |
| H200002156 |  | 1.24 |  |  | 0.0074 |  |  | [AK054689](http://www.ncbi.nlm.nih.gov/entrez/query.fcgi?db=Nucleotide&term=AK054689,BC022544) |  |
| H200002632 | Y | 0.77 | 1.14 |  | 0.0018 |  |  | [D86964](http://www.ncbi.nlm.nih.gov/entrez/query.fcgi?db=Nucleotide&term=D86964) | [DOCK2](http://www.ncbi.nlm.nih.gov/entrez/query.fcgi?cmd=search&db=gene&term=DOCK2) |
| H200002708 | Y | 0.75 | 1.00 |  | 0.0015 | 0.9819 |  | [NM_005558](http://www.ncbi.nlm.nih.gov/entrez/query.fcgi?db=Nucleotide&term=BC009742,U42408) | [LAD1](http://www.ncbi.nlm.nih.gov/entrez/query.fcgi?cmd=search&db=gene&term=LAD1) |
| H200002716 | Y | 0.69 | 1.46 |  | 0.0052 |  |  | [NM_012093](http://www.ncbi.nlm.nih.gov/entrez/query.fcgi?db=Nucleotide&term=AY171600) | [AK5](http://www.ncbi.nlm.nih.gov/entrez/query.fcgi?cmd=search&db=gene&term=AK5) |
| H200002784 |  | 0.79 |  |  | 0.0088 |  |  | [AB037725](http://www.ncbi.nlm.nih.gov/entrez/query.fcgi?db=Nucleotide&term=BC053903) | [SRGAP1](http://www.ncbi.nlm.nih.gov/entrez/query.fcgi?cmd=search&db=gene&term=SRGAP1) |
| **Unique id** | **Spira set** | **Mean array fold change** | **Mean TPCH training fold change** | **Mean TPCH independent set fold change** | **Array Parametric p-value** | **TPCH training p- value** | **TPCH independent set p- value** | **GeneBank accession** | **Gene symbol** |
| H200002856 | Y | 0.66 | 1.02 |  | 0.0003 |  |  | [NM_030793](http://www.ncbi.nlm.nih.gov/entrez/query.fcgi?db=Nucleotide&term=BC033454) | [SP329](http://www.ncbi.nlm.nih.gov/entrez/query.fcgi?cmd=search&db=gene&term=FBXO38) |
| H200003203 | Y | 0.56 | 1.22 |  | 0.0001 |  |  | [NM_005700](http://www.ncbi.nlm.nih.gov/entrez/query.fcgi?db=Nucleotide&term=AL833475,AJ271216) | [DPP3](http://www.ncbi.nlm.nih.gov/entrez/query.fcgi?cmd=search&db=gene&term=DPP3) |
| H200003282 |  | 1.43 |  |  | 0.0019 |  |  | [NM_018842](http://www.ncbi.nlm.nih.gov/entrez/query.fcgi?db=Nucleotide&term=) | [LOC55971](http://www.ncbi.nlm.nih.gov/entrez/query.fcgi?cmd=search&db=gene&term=NP_061330) |
| H200003420 | Y | 1.29 | 1.37 |  | 0.0082 | 0.1024 |  | [NM_022136](http://www.ncbi.nlm.nih.gov/entrez/query.fcgi?db=Nucleotide&term=AF218085) | [SAMSN1](http://www.ncbi.nlm.nih.gov/entrez/query.fcgi?cmd=search&db=gene&term=SAMSN1) |
| H200003440 | Y | 1.38 | * |  | 0.0010 |  |  | [AK056630](http://www.ncbi.nlm.nih.gov/entrez/query.fcgi?db=Nucleotide&term=AK056630) |  |
| H200003537 | Y | 0.75 | 0.99 |  | 0.0051 | 0.9492 |  | [NM_001330](http://www.ncbi.nlm.nih.gov/entrez/query.fcgi?db=Nucleotide&term=U43030,BC036787) | [CTF1](http://www.ncbi.nlm.nih.gov/entrez/query.fcgi?cmd=search&db=gene&term=CTF1) |
| H200003640 | Y | 0.76 | 1.15 |  | 0.0067 |  |  | [NM_017782](http://www.ncbi.nlm.nih.gov/entrez/query.fcgi?db=Nucleotide&term=AK000367,BX649177) | [FLJ20360](http://www.ncbi.nlm.nih.gov/entrez/query.fcgi?cmd=search&db=gene&term=Q8IVG4) |
| H200004155 |  | 0.73 |  |  | 0.0044 |  |  | [NM_033317](http://www.ncbi.nlm.nih.gov/entrez/query.fcgi?db=Nucleotide&term=) | [ZD52F10](http://www.ncbi.nlm.nih.gov/entrez/query.fcgi?cmd=search&db=gene&term=) |
| H200004928 | Y | 1.36 | 1.05 |  | 0.0090 | 0.6990 |  | [NM_003136](http://www.ncbi.nlm.nih.gov/entrez/query.fcgi?db=Nucleotide&term=BC000652,U51920) | [SRP54](http://www.ncbi.nlm.nih.gov/entrez/query.fcgi?cmd=search&db=gene&term=SRP54) |
| H200005125 |  | 0.54 |  |  | 0.0089 |  |  | [NM_054023](http://www.ncbi.nlm.nih.gov/entrez/query.fcgi?db=Nucleotide&term=BG484055) |  |
| H200005339 | Y | 0.64 | 1.16 |  | 0.0003 |  |  | [NM_013352](http://www.ncbi.nlm.nih.gov/entrez/query.fcgi?db=Nucleotide&term=BC039245) | [SART-2](http://www.ncbi.nlm.nih.gov/entrez/query.fcgi?cmd=search&db=gene&term=SART2) |
| H200005357 | Y | 0.77 | 0.98 |  | 0.0093 | 0.9236 |  | [NM_014037](http://www.ncbi.nlm.nih.gov/entrez/query.fcgi?db=Nucleotide&term=BC034948) | [NTT5](http://www.ncbi.nlm.nih.gov/entrez/query.fcgi?cmd=search&db=gene&term=SLC6A16) |
| H200005448 | Y | 1.26 | 1.15 |  | 0.0041 | 0.1839 |  | [NM_012334](http://www.ncbi.nlm.nih.gov/entrez/query.fcgi?db=Nucleotide&term=AB018342,AF234532) | [MYO10](http://www.ncbi.nlm.nih.gov/entrez/query.fcgi?cmd=search&db=gene&term=MYO10) |
| H200005581 |  | 1.30 |  |  | 0.0087 |  |  | [AF339776](http://www.ncbi.nlm.nih.gov/entrez/query.fcgi?db=Nucleotide&term=AF339776) |  |
| H200006290 | Y | 1.64 | 7.53 | 0.39 | 0.0049 | 0.0194 |  | [NM_000300](http://www.ncbi.nlm.nih.gov/entrez/query.fcgi?db=Nucleotide&term=M22430) | [PLA2G2A](http://www.ncbi.nlm.nih.gov/entrez/query.fcgi?cmd=search&db=gene&term=PLA2G2A) |
| H200006635 | Y | 1.42 | 2.05 | 1.14 | 0.0098 | 0.0224 | 0.7471 | [NM_001796](http://www.ncbi.nlm.nih.gov/entrez/query.fcgi?db=Nucleotide&term=AK124734,AB035305) | [CDH8](http://www.ncbi.nlm.nih.gov/entrez/query.fcgi?cmd=search&db=gene&term=CDH8) |
| H200006810 | Y | 1.44 | 1.79 | 2.90 | 0.0016 | 0.0140 | 0.0867 | [NM_004369](http://www.ncbi.nlm.nih.gov/entrez/query.fcgi?db=Nucleotide&term=X52022) | [COL6A3](http://www.ncbi.nlm.nih.gov/entrez/query.fcgi?cmd=search&db=gene&term=COL6A3) |
| H200007262 | Y | 1.33 | 1.16 |  | 0.0008 | 0.4523 |  | [L22650](http://www.ncbi.nlm.nih.gov/entrez/query.fcgi?db=Nucleotide&term=L22650) | [EPAG](http://www.ncbi.nlm.nih.gov/entrez/query.fcgi?cmd=search&db=gene&term=Q14236) |
| H200007273 |  | 1.26 |  |  | 0.0031 |  |  | [BC011630](http://www.ncbi.nlm.nih.gov/entrez/query.fcgi?db=Nucleotide&term=BC011630) |  |
| H200007497 |  | 1.47 |  |  | 0.0013 |  |  | [AB037804](http://www.ncbi.nlm.nih.gov/entrez/query.fcgi?db=Nucleotide&term=AB037804) | [KIAA1383](http://www.ncbi.nlm.nih.gov/entrez/query.fcgi?cmd=search&db=gene&term=YD83_HUMAN) |
| H200007517 | Y | 0.81 | 1.06 |  | 0.0086 |  |  | [NM_014908](http://www.ncbi.nlm.nih.gov/entrez/query.fcgi?db=Nucleotide&term=AB029017,BC035556) | [KIAA1094](http://www.ncbi.nlm.nih.gov/entrez/query.fcgi?cmd=search&db=gene&term=TMEM15) |
| H200007642 | Y | 1.26 | 1.16 |  | 0.0100 | 0.3266 |  | [NM_015640](http://www.ncbi.nlm.nih.gov/entrez/query.fcgi?db=Nucleotide&term=BM827226,AK074970,BC003049,AL080119) | [PAI-RBP1](http://www.ncbi.nlm.nih.gov/entrez/query.fcgi?cmd=search&db=gene&term=NP_056455) |
| H200007690 |  | 0.76 |  |  | 0.0017 |  |  | [AK025893](http://www.ncbi.nlm.nih.gov/entrez/query.fcgi?db=Nucleotide&term=AK025893) |  |
| H200007838 |  | 1.32 |  |  | 0.0051 |  |  | [AB051548](http://www.ncbi.nlm.nih.gov/entrez/query.fcgi?db=Nucleotide&term=AB051548,AL834196) | [FLJ14743](http://www.ncbi.nlm.nih.gov/entrez/query.fcgi?cmd=search&db=gene&term=FAM40A) |
| H200007867 | Y | 1.44 | 1.30 |  | 0.0021 | 0.2526 |  | [NM_003318](http://www.ncbi.nlm.nih.gov/entrez/query.fcgi?db=Nucleotide&term=BC000633) | [TTK](http://www.ncbi.nlm.nih.gov/entrez/query.fcgi?cmd=search&db=gene&term=TTK) |
| H200008070 | Y | 1.40 | 0.84 |  | 0.0031 |  |  | [NM_002183](http://www.ncbi.nlm.nih.gov/entrez/query.fcgi?db=Nucleotide&term=BC035407) | [IL3RA](http://www.ncbi.nlm.nih.gov/entrez/query.fcgi?cmd=search&db=gene&term=IL3RA) |
| H200008154 | Y | 1.24 | 1.54 | 2.50 | 0.0099 | 0.0323 | 0.1203 | [NM_002615](http://www.ncbi.nlm.nih.gov/entrez/query.fcgi?db=Nucleotide&term=) | [SERPINF1](http://www.ncbi.nlm.nih.gov/entrez/query.fcgi?cmd=search&db=gene&term=SERPINF1) |
| H200008156 | Y | 1.26 | 1.01 |  | 0.0051 | 0.9382 |  | [NM_014904](http://www.ncbi.nlm.nih.gov/entrez/query.fcgi?db=Nucleotide&term=AB023158) | [Rab11-FIP2](http://www.ncbi.nlm.nih.gov/entrez/query.fcgi?cmd=search&db=gene&term=RAB11FIP2) |
| H200008288 |  | 0.75 |  |  | 0.0043 |  |  | [AK057131](http://www.ncbi.nlm.nih.gov/entrez/query.fcgi?db=Nucleotide&term=BC063477,AK057131) |  |
| H200008433 | Y | 0.75 | 1.06 |  | 0.0055 |  |  | [NM_017512](http://www.ncbi.nlm.nih.gov/entrez/query.fcgi?db=Nucleotide&term=X67098,AK127219) | [HSRTSBETA](http://www.ncbi.nlm.nih.gov/entrez/query.fcgi?cmd=search&db=gene&term=NP_974487) |
| H200008505 | Y | 0.66 | 1.65 |  | 0.00003 |  |  | [NM_004306](http://www.ncbi.nlm.nih.gov/entrez/query.fcgi?db=Nucleotide&term=Z11502,AJ306450) | [ANXA13](http://www.ncbi.nlm.nih.gov/entrez/query.fcgi?cmd=search&db=gene&term=ANXA13) |
| **Unique id** | **Spira set** | **Mean array fold change** | **Mean TPCH training fold change** | **Mean TPCH independent set fold change** | **Array Parametric p-value** | **TPCH training p- value** | **TPCH independent set p- value** | **GeneBank accession** | **Gene symbol** |
| H200008939 |  | 1.37 |  |  | 0.0022 |  |  | [AK021603](http://www.ncbi.nlm.nih.gov/entrez/query.fcgi?db=Nucleotide&term=) |  |
| H200008958 |  | 1.46 |  |  | 0.0090 |  |  | [AK021793](http://www.ncbi.nlm.nih.gov/entrez/query.fcgi?db=Nucleotide&term=AK096022,AK021793) |  |
| H200008994 |  | 1.58 |  |  | 0.0098 |  |  | [AK022258](http://www.ncbi.nlm.nih.gov/entrez/query.fcgi?db=Nucleotide&term=AK022258) |  |
| H200009299 | Y | 0.60 | 0.60 | 1.38 | 0.0001 | 0.0073 |  | [NM_002644](http://www.ncbi.nlm.nih.gov/entrez/query.fcgi?db=Nucleotide&term=X73079,CR749533) | [PIGR](http://www.ncbi.nlm.nih.gov/entrez/query.fcgi?cmd=search&db=gene&term=PIGR) |
| H200009441 | Y | 0.77 | 0.87 |  | 0.0034 | 0.4056 |  | [NM_020672](http://www.ncbi.nlm.nih.gov/entrez/query.fcgi?db=Nucleotide&term=BG674026) | [LOC57402](http://www.ncbi.nlm.nih.gov/entrez/query.fcgi?cmd=search&db=gene&term=S100A14) |
| H200010090 | Y | 1.32 | 1.11 |  | 0.0002 | 0.1548 |  | [AF084555](http://www.ncbi.nlm.nih.gov/entrez/query.fcgi?db=Nucleotide&term=AL833077,AF084555) | [ARPP-19](http://www.ncbi.nlm.nih.gov/entrez/query.fcgi?cmd=search&db=gene&term=AP19_HUMAN) |
| H200010806 |  | 1.27 |  |  | 0.0038 |  |  | [AK057432](http://www.ncbi.nlm.nih.gov/entrez/query.fcgi?db=Nucleotide&term=AK057432) |  |
| H200011062 |  | 0.77 |  |  | 0.0081 |  |  | [AB067467](http://www.ncbi.nlm.nih.gov/entrez/query.fcgi?db=Nucleotide&term=BX537534,AB067467) | [KIAA1880](http://www.ncbi.nlm.nih.gov/entrez/query.fcgi?cmd=search&db=gene&term=) |
| H200011229 |  | 1.32 |  |  | 0.0008 |  |  | [AK024978](http://www.ncbi.nlm.nih.gov/entrez/query.fcgi?db=Nucleotide&term=AK024978) | [LSR68](http://www.ncbi.nlm.nih.gov/entrez/query.fcgi?cmd=search&db=gene&term=C14orf43) |
| H200011233 | Y | 1.34 | 1.35 | 1.44 | 0.0088 | 0.0385 | 0.3385 | [NM_016588](http://www.ncbi.nlm.nih.gov/entrez/query.fcgi?db=Nucleotide&term=AF136631,BC042019) | [LOC51299](http://www.ncbi.nlm.nih.gov/entrez/query.fcgi?cmd=search&db=gene&term=NRN1) |
| H200011369 | Y | 1.33 | 1.13 |  | 0.0083 | 0.3644 |  | [NM_005264](http://www.ncbi.nlm.nih.gov/entrez/query.fcgi?db=Nucleotide&term=AF038421) | [GFRA1](http://www.ncbi.nlm.nih.gov/entrez/query.fcgi?cmd=search&db=gene&term=GFRA1) |
| H200011944 | Y | 1.33 | 1.01 |  | 0.0002 |  |  | [NM_002618](http://www.ncbi.nlm.nih.gov/entrez/query.fcgi?db=Nucleotide&term=AF048755) | [PEX13](http://www.ncbi.nlm.nih.gov/entrez/query.fcgi?cmd=search&db=gene&term=PEX13) |
| H200012231 |  | 1.28 |  |  | 0.0092 |  |  | [AL117477](http://www.ncbi.nlm.nih.gov/entrez/query.fcgi?db=Nucleotide&term=BX640713,AL117477) | [DKFZP727G051](http://www.ncbi.nlm.nih.gov/entrez/query.fcgi?cmd=search&db=gene&term=PHF19) |
| H200012824 |  | 0.81 |  |  | 0.0069 |  |  | [NM_024762](http://www.ncbi.nlm.nih.gov/entrez/query.fcgi?db=Nucleotide&term=) | [FLJ21603](http://www.ncbi.nlm.nih.gov/entrez/query.fcgi?cmd=search&db=gene&term=) |
| H200013205 |  | 1.30 |  |  | 0.0088 |  |  | [AL137315](http://www.ncbi.nlm.nih.gov/entrez/query.fcgi?db=Nucleotide&term=BX537972,AL137315) |  |
| H200013655 |  | 0.80 |  |  | 0.0085 |  |  | [AF119843](http://www.ncbi.nlm.nih.gov/entrez/query.fcgi?db=Nucleotide&term=) | [PPP4R1L](http://www.ncbi.nlm.nih.gov/entrez/query.fcgi?cmd=search&db=gene&term=PPP4R1L) |
| H200014078 |  | 0.79 |  |  | 0.0031 |  |  | [NM_005339](http://www.ncbi.nlm.nih.gov/entrez/query.fcgi?db=Nucleotide&term=CR605719,U58522) | [HIP2](http://www.ncbi.nlm.nih.gov/entrez/query.fcgi?cmd=search&db=gene&term=UBC1_HUMAN) |
| H200014189 | Y | 0.76 | * |  | 0.0077 |  |  | [NM_000200](http://www.ncbi.nlm.nih.gov/entrez/query.fcgi?db=Nucleotide&term=M26665,AK130505) | [HTN3](http://www.ncbi.nlm.nih.gov/entrez/query.fcgi?cmd=search&db=gene&term=) |
| H200014801 |  | 1.24 |  |  | 0.0084 |  |  | [NM_021105](http://www.ncbi.nlm.nih.gov/entrez/query.fcgi?db=Nucleotide&term=AB006746) | [PLSCR1](http://www.ncbi.nlm.nih.gov/entrez/query.fcgi?cmd=search&db=gene&term=PLSCR1) |
| H200015239 |  | 1.34 |  |  | 0.0026 |  |  | [NM_032365](http://www.ncbi.nlm.nih.gov/entrez/query.fcgi?db=Nucleotide&term=CR749832) | [MGC5254](http://www.ncbi.nlm.nih.gov/entrez/query.fcgi?cmd=search&db=gene&term=NP_054828) |
| H200015337 | Y | 1.26 | * |  | 0.0060 |  |  | [AL109681](http://www.ncbi.nlm.nih.gov/entrez/query.fcgi?db=Nucleotide&term=AL109681) |  |
| H200015361 |  | 0.81 |  |  | 0.0098 |  |  | [U50748](http://www.ncbi.nlm.nih.gov/entrez/query.fcgi?db=Nucleotide&term=U50748) | [LEPR](http://www.ncbi.nlm.nih.gov/entrez/query.fcgi?cmd=search&db=gene&term=) |
| H200015466 | Y | 0.75 | * |  | 0.0066 |  |  | [NM_017928](http://www.ncbi.nlm.nih.gov/entrez/query.fcgi?db=Nucleotide&term=AK000701) | [FLJ20694](http://www.ncbi.nlm.nih.gov/entrez/query.fcgi?cmd=search&db=gene&term=NP_060398) |
| H200015598 |  | 0.78 |  |  | 0.0048 |  |  | [AK022114](http://www.ncbi.nlm.nih.gov/entrez/query.fcgi?db=Nucleotide&term=AK022114,BX648730) |  |
| H200016125 |  | 1.26 |  |  | 0.0099 |  |  | [X57131](http://www.ncbi.nlm.nih.gov/entrez/query.fcgi?db=Nucleotide&term=) | [H2AFFP](http://www.ncbi.nlm.nih.gov/entrez/query.fcgi?cmd=search&db=gene&term=) |
| H200016317 | Y | 0.74 | 0.78 | 1.13 | 0.0006 | 0.0251 |  | [NM_002999](http://www.ncbi.nlm.nih.gov/entrez/query.fcgi?db=Nucleotide&term=BC030805) | [SDC4](http://www.ncbi.nlm.nih.gov/entrez/query.fcgi?cmd=search&db=gene&term=SDC4) |
| H200016589 |  | 1.43 |  |  | 0.00001 |  |  | [AB037830](http://www.ncbi.nlm.nih.gov/entrez/query.fcgi?db=Nucleotide&term=AB037830) | [KIAA1409](http://www.ncbi.nlm.nih.gov/entrez/query.fcgi?cmd=search&db=gene&term=KIAA1409) |
| H200016590 | Y | 0.78 | 0.93 |  | 0.0023 | 0.6033 |  | [NM_016569](http://www.ncbi.nlm.nih.gov/entrez/query.fcgi?db=Nucleotide&term=AF216750) | [TBX3](http://www.ncbi.nlm.nih.gov/entrez/query.fcgi?cmd=search&db=gene&term=TBX3) |
| H200016675 |  | 1.28 |  |  | 0.0064 |  |  | [BC014124](http://www.ncbi.nlm.nih.gov/entrez/query.fcgi?db=Nucleotide&term=BC014124,BC022089) |  |
| H200017180 |  | 0.80 |  |  | 0.0073 |  |  | [AL157439](http://www.ncbi.nlm.nih.gov/entrez/query.fcgi?db=Nucleotide&term=AL157439) |  |
| H200017197 | Y | 1.55 | 1.21 |  | 0.0031 | 0.2468 |  | [NM_005525](http://www.ncbi.nlm.nih.gov/entrez/query.fcgi?db=Nucleotide&term=) | [HSD11B1](http://www.ncbi.nlm.nih.gov/entrez/query.fcgi?cmd=search&db=gene&term=HSD11B1) |
| **Unique id** | **Spira set** | **Mean array fold change** | **Mean TPCH training fold change** | **Mean TPCH independent set fold change** | **Array Parametric p-value** | **TPCH training p- value** | **TPCH independent set p- value** | **GeneBank accession** | **Gene symbol** |
| H200017277 | Y | 0.71 | * |  | 0.0031 |  |  | [NM_000500](http://www.ncbi.nlm.nih.gov/entrez/query.fcgi?db=Nucleotide&term=CD013987) | [CYP21A2](http://www.ncbi.nlm.nih.gov/entrez/query.fcgi?cmd=search&db=gene&term=CYP21A2) |
| H200018561 |  | 1.31 |  |  | 0.0005 |  |  | [AK025148](http://www.ncbi.nlm.nih.gov/entrez/query.fcgi?db=Nucleotide&term=AK025148) |  |
| H200018762 |  | 1.27 |  |  | 0.0036 |  |  | [NM_032135](http://www.ncbi.nlm.nih.gov/entrez/query.fcgi?db=Nucleotide&term=BC039878) | [DKFZP434F1017](http://www.ncbi.nlm.nih.gov/entrez/query.fcgi?cmd=search&db=gene&term=C14orf155) |
| H200018789 |  | 1.31 |  |  | 0.0074 |  |  | [U79276](http://www.ncbi.nlm.nih.gov/entrez/query.fcgi?db=Nucleotide&term=U79276) |  |
| H200018824 |  | 0.79 |  |  | 0.0074 |  |  | [AK001567](http://www.ncbi.nlm.nih.gov/entrez/query.fcgi?db=Nucleotide&term=BC042043,AK001567) |  |
| H200019126 |  | 1.29 |  |  | 0.0057 |  |  | [AK054633](http://www.ncbi.nlm.nih.gov/entrez/query.fcgi?db=Nucleotide&term=AK054633) | [IMAGE3451454](http://www.ncbi.nlm.nih.gov/entrez/query.fcgi?cmd=search&db=gene&term=NP_444280) |
| H200019211 |  | 1.34 |  |  | 0.0078 |  |  | [AF339828](http://www.ncbi.nlm.nih.gov/entrez/query.fcgi?db=Nucleotide&term=AF339828) |  |
| H200019309 |  | 1.27 |  |  | 0.0029 |  |  | [NM_032546](http://www.ncbi.nlm.nih.gov/entrez/query.fcgi?db=Nucleotide&term=AJ291714) | [RNF30](http://www.ncbi.nlm.nih.gov/entrez/query.fcgi?cmd=search&db=gene&term=RNF30) |
| H200019452 |  | 1.28 |  |  | 0.0087 |  |  | [AK023292](http://www.ncbi.nlm.nih.gov/entrez/query.fcgi?db=Nucleotide&term=AK023292) |  |
| H200019497 |  | 1.38 |  |  | 0.0045 |  |  | [AK025983](http://www.ncbi.nlm.nih.gov/entrez/query.fcgi?db=Nucleotide&term=AK025983) |  |
| H200019564 |  | 1.37 |  |  | 0.0061 |  |  | [NM_033062](http://www.ncbi.nlm.nih.gov/entrez/query.fcgi?db=Nucleotide&term=AJ406934) | [KAP4.2](http://www.ncbi.nlm.nih.gov/entrez/query.fcgi?cmd=search&db=gene&term=) |
| H200019565 |  | 0.81 |  |  | 0.0079 |  |  | [BC017742](http://www.ncbi.nlm.nih.gov/entrez/query.fcgi?db=Nucleotide&term=BC017742,BX537363) |  |
| H200020785 |  | 1.27 |  |  | 0.0085 |  |  | [AK056398](http://www.ncbi.nlm.nih.gov/entrez/query.fcgi?db=Nucleotide&term=AK056398) |  |
| H200020805 |  | 1.52 |  |  | 0.0088 |  |  | [AK056109](http://www.ncbi.nlm.nih.gov/entrez/query.fcgi?db=Nucleotide&term=BC039073,AK056109) |  |
| H200020945 |  | 0.79 |  |  | 0.0097 |  |  | [AK054715](http://www.ncbi.nlm.nih.gov/entrez/query.fcgi?db=Nucleotide&term=) |  |
| H200021147 |  | 1.28 |  |  | 0.0021 |  |  | [AK057241](http://www.ncbi.nlm.nih.gov/entrez/query.fcgi?db=Nucleotide&term=BC066774,AK057241,BC063309) |  |
| H200021295 |  | 0.80 |  |  | 0.0044 |  |  | [BC017706](http://www.ncbi.nlm.nih.gov/entrez/query.fcgi?db=Nucleotide&term=) |  |

**Probes that blasted partly or fully to multiple gene sequences, **DNA sequences difficult to design primers and /or optimize*
