# Supplementary material for: Expression profiling identifies genes involved in emphysema severity
Source: Respir Res. 2009 Sep 2;10(1):81. doi: 10.1186/1465-9921-10-81 (PMC2746189; doi:10.1186/1465-9921-10-81)
Supplement: Additional file 3 — Comparison of class prediction analysis of 51 genes in public datasets. Class prediction results of 51 genes in TPCH training, Spira and Golpon dataset using Nearest Centroid Correct algorithm. "YES" indicates that the sample has been classified correctly and "NO" indicates that the sample has been classified incorrectly. [file 1465-9921-10-81-S3.doc]

**Additional file 3**

**File Format:** DOC

**Title:** Comparison of class prediction analysis of 51 genes in public datasets.

**Description:** Class prediction results of 51 genes in TPCH training, Spira and Golpon dataset using Nearest Centroid Correct algorithm. “YES” indicates that the sample has been classified correctly and “NO” indicates that the sample has been classified incorrectly.

| **TPCH samples** | **TPCH training** | **Spira Samples** | **Spira** | **Golpon samples** | **Golpon** |
| --- | --- | --- | --- | --- | --- |
| GSM443033 | YES | GSM28357 | YES | GSM18413 | YES |
| GSM443035 | YES | GSM28359 | YES | GSM18414 | YES |
| GSM443036 | YES | GSM28361 | YES | GSM18415 | YES |
| GSM443038 | YES | GSM28363 | YES | GSM18416 | YES |
| GSM443039 | YES | GSM28364 | YES | GSM18417 | NO |
| GSM443040 | YES | GSM28365 | YES | GSM18403 | YES |
| GSM443041 | YES | GSM28366 | NO | GSM18404 | YES |
| GSM443042 | YES | GSM28367 | YES | GSM18405 | YES |
| GSM443044 | YES | GSM28368 | YES | GSM18406 | NO |
| GSM443045 | YES | GSM28369 | NO | GSM18407 | YES |
| GSM443046 | YES | GSM28371 | YES | - | - |
| GSM443048 | YES | GSM28373 | NO | - | - |
| GSM443049 | YES | GSM28375 | YES | - | - |
| GSM443051 | YES | GSM28377 | YES | - | - |
| GSM443053 | YES | GSM28379 | YES | - | - |
| GSM443054 | YES | GSM28381 | YES | - | - |
| GSM443055 | YES | GSM28383 | YES | - | - |
| GSM443056 | YES | GSM28385 | YES | - | - |
| GSM443057 | YES | GSM28358 | YES | - | - |
| GSM443060 | YES | GSM28360 | YES | - | - |
| GSM442993 | YES | GSM28362 | YES | - | - |
| GSM443033 | YES | GSM28370 | NO | - | - |
| GSM442624 | YES | GSM28372 | YES | - | - |
| GSM443037 | YES | GSM28374 | NO | - | - |
| GSM443043 | YES | GSM28376 | YES | - | - |
| GSM443047 | YES | GSM28378 | NO | - | - |
| GSM443050 | YES | GSM28380 | YES | - | - |
| GSM443052 | YES | GSM28382 | NO | - | - |
| GSM443058 | YES | GSM28384 | YES | - | - |
| GSM443059 | YES | GSM28386 | YES | - | - |
|  | 100 |  | 77 |  | 80 |
