# Supplementary material for: Expression profiling identifies genes involved in emphysema severity
Source: Respir Res. 2009 Sep 2;10(1):81. doi: 10.1186/1465-9921-10-81 (PMC2746189; doi:10.1186/1465-9921-10-81)
Supplement: Additional file 4 — Dendrogram of shortlisted 51 genes. Supervised two-dimensional hierarchical clustering based on average linkage uncentered correlation of emphysema samples using microarray expression data of the 51 genes represented in Spira and Golpon platforms chosen for qRT-PCR validation on TPCH training set. Each column represents a sample and each row represents a gene. Mild emphysema samples are indicated by the blue bar and moderate emphysema samples are indicated by the orange bar. Heatmap indicates level of gene expression, red, high expression, green, low expression in moderate compared to mild emphysema severity. [file 1465-9921-10-81-S4.doc]

**Additional file 4**

**File format:** DOC

**Title:** Dendrogram of shortlisted 51 genes.

**Description:** Supervised two-dimensional hierarchical clustering based on average linkage uncentered correlation of emphysema samples using microarray expression data of the 51 genes represented in Spira and Golpon platforms chosen for qRT-PCR validation on TPCH training set. Each column represents a sample and each row represents a gene. Mild emphysema samples are indicated by the blue bar and moderate emphysema samples are indicated by the orange bar. Heatmap indicates level of gene expression, red, high expression, green, low expression in moderate compared to mild emphysema severity.
