# Supplementary material for: Expression profiling identifies genes involved in emphysema severity
Source: Respir Res. 2009 Sep 2;10(1):81. doi: 10.1186/1465-9921-10-81 (PMC2746189; doi:10.1186/1465-9921-10-81)
Supplement: Additional file 6 — Pathway analysis on candidate genes. Canonical Pathway analysis in IPA on the seven validated candidate genes. The most significant functional and canonical groups, with p < 0.05 are presented. The bars represent p-value in logarithmic scale for each functional or canonical group and genes assigned to each of the functions are listed. [file 1465-9921-10-81-S6.doc]

**Additional file 6**

**File Format:** DOC

**Title:** Pathway analysis on candidate genes

**Description:**  Canonical Pathway analysis in IPA on the seven validated candidate genes. The most significant functional and canonical groups, with *p* <0.05 are presented. The bars represent p-value in logarithmic scale for each functional or canonical group and genes assigned to each of the functions are listed.
