# Supplementary material for: Expression profiling identifies genes involved in emphysema severity
Source: Respir Res. 2009 Sep 2;10(1):81. doi: 10.1186/1465-9921-10-81 (PMC2746189; doi:10.1186/1465-9921-10-81)
Supplement: Additional file 7 — Over-representation of gene ontologies in candidate genes. Heatmap (a) and enrichment score (b) of gene ontologies overrepresented in six of the seven candidates. a) Represents common gene ontologies enriched in the candidate genes. b) Significant clustering (Fisher's Exact, p < 0.05) of molecular, biological and cellular functions in the candidate genes. [file 1465-9921-10-81-S7.doc]

**Additional file 7**

**File Format:** DOC

**Title:** Over-representation of gene ontologies in candidate genes

**Description:** Heatmap (a) and enrichment score (b) of gene ontologies overrepresented in six of the seven candidates.  **a)** Represents common gene ontologies enriched in the candidate genes. **b)** Significant clustering (Fisher’s Exact, *p* <0.05) of molecular, biological and cellular functions in the candidate genes.

**7a**

**7b**
